# Diversity in Advance Care Planning and End-Of-Life Conversations: Discourses of Healthcare Professionals and Researchers

**DOI:** 10.1177/00302228221126257

**Published:** 2022-09-13

**Authors:** Charlotte Kröger, Özgül Uysal-Bozkir, Mike J. L. Peters, Annicka G. M. Van der Plas, Guy A. M. Widdershoven, Maaike E. Muntinga

**Affiliations:** 1 Department of Ethics, Law and Humanities, 1209 Amsterdam UMC, Vrije Universiteit Amsterdam, Amsterdam, The Netherlands; 2 Department of Internal and Geriatric Medicine, 1209 Amsterdam UMC, Universiteit van Amsterdam, Amsterdam, The Netherlands; 3 Department of Public and Occupational Health, 1209 Amsterdam UMC, Vrije Universiteit Amsterdam, Amsterdam, The Netherlands

**Keywords:** diversity, discourses, end-of-life care, end-of-life conversations, advance care planning, equity

## Abstract

To meet the end-of-life needs of all patients, ongoing conversations about values and preferences regarding end-of-life care are essential. Aspects of social identity are associated with disparities in end-of-life care outcomes. Therefore, accounting for patient diversity in advance care planning and end-of-life conversations is important for equitable end-of-life practices. We conducted 16 semi-structured interviews to explore how Dutch healthcare professionals and researchers conceptualized diversity in advance care planning and end-of-life conversations and how they envision diversity-responsive end-of-life care and research. Using thematic discourse analysis, we identified five ‘diversity discourses’: the categorical discourse; the diversity as a determinant discourse; the diversity in norms and values discourse; the everyone is unique discourse, and the anti-essentialist discourse. These discourses may have distinct implications for diversity-responsive end-of-life conversations, care and research. Awareness and reflection on these discourses may contribute to more inclusive end-of-life practices.

## Introduction

Talking about the end-of-life is an intricate and highly personal endeavor. Not only do ideas about what a good life and death is vary across individuals and cultural groups, but there are also different moral perspectives on which care interventions could facilitate such a good death. Aspects of social identity and diversity influence preferences, values and needs at the end-of-life, and have been associated with health and care disparities, including disparities in end-of-life care (Barrett & Wholihan, 2016; Cloyes et al., 2018; Corpora, 2021; Grindrod, 2020; Ha et al., 2021; Napier et al., 2014; De Vries et al., 2019). Therefore, accounting for diversity in conversations about the end-of-life is key to provide inclusive and equity-oriented care. Since everyday health care and its context is shaped by research-informed standards of care and policy directives, the extent to and manner in which diversity is considered throughout every step of the research-to-practice cycle might inform the everyday implementation of inclusive end-of-life practices. In this explorative qualitative study, we report results of a thematic discourse analysis to investigate how healthcare professionals and researchers in the Netherlands conceptualize and envision diversity in advance care planning (ACP) and end-of-life conversations.

### End-of-Life Conversations and Advance Care Planning

Ongoing conversations about end-of-life care between patients, important people in their lives and healthcare professionals are key to reflect on fundamental and often sensitive questions around future and end-of-life care, and to explicate and discuss values and wishes (Fulmer et al., 2018; Kwak & Haley, 2005). Multiple approaches to such conversations exist. ‘End-of-life conversations’ is a more general term to describe all end-of-life care communication, which can happen before or during the last phase of life, while the term ‘ACP’ specifically refers to a systematically implemented form of these conversations. So, while end-of-life conversations can occur before and *at* the end-of-life, ACP is a process specifically aimed at defining *future* care that is consistent with a person’s preferences, including situations when someone is no longer able to make their own decisions (Detering et al., 2010; Sudore et al., 2017). Across both, interventions, approaches and contexts vary (Goswami, 2021). Conversations about the end-of-life can entail a variety of emotionally laden subjects, including treatment choices, treatment failure, unfavorable prognoses, anticipatory mourning, family responses, concerns about coping, life goals, perspectives on life-sustaining treatment, and about the meaning of illness, death and suffering (Larson & Tobin, 2000). The ACP process can specifically include choosing a surrogate decision-maker or formulating an advance directive and should be formerly recorded (Detering et al., 2010; Sudore et al., 2017).

Literature suggests that communication about end-of-life wishes, for instance by engaging in a good ACP process, can improve care experiences, quality of end-of-life care and care outcomes (Brinkman-Stoppelenburg et al., 2014; Detering et al., 2010; Van der Plas et al., 2021). Hence, promoting and normalizing ACP and end-of-life conversations as a way to enhance quality of care for all, is an important (public) health issue (Gallagher et al., 2020; Prince-Paul & DiFranco, 2017) that requires understanding and accounting for interpersonal variation in care, research and policy.

### Disparities in End-of-Life Care and Communication

End-of-life care takes place in a life phase in which diversity-related factors like value systems, knowledge and experiences determine quality of and access to services; at the same time, increasing societal and moral pluralism has contributed to a health care context in which a growing variation of such factors shape how good end-of-life care is understood, valued, and delivered (Hong & Kim, 2020; Johnstone & Kanitsaki, 2009; Portanova et al., 2017; Seelman et al., 2018; Volandes et al., 2008; Beringer et al., 2021; Napier et al., 2014).

There has been mounting evidence that aspects of social identity (such as culture and ethnicity, class, religion or spirituality, and sexual and gender identity) influence outcomes of end-of-life conversations and ACP (Barrett & Wholihan, 2016; Cloyes et al., 2018; Corpora, 2021; Grindrod, 2020; Ha et al., 2021; De Vries et al., 2019). Research suggests that socio-structural factors impact the way in which end-of-life choices, and thus opportunities for good end-of-life care, are distributed in society (Bloomer & Walshe, 2021; Davies et al., 2021; Grindrod, 2020; Inoue, 2016). Indeed, inequities between various minoritized and majoritized patient populations have been well-documented in studies on end-of-life care, decision-making and ACP across various axes of social and cultural difference (Cloyes et al., 2018; Evans & Ume, 2011; Kwak & Haley, 2005; Sue & Dhindsa, 2006; Torensma et al., 2020). For instance, patients with ethnically minoritized backgrounds less often access and complete ACP (Portanova et al., 2017). Additionally, aspects like health literacy, language proficiency, non-verbal communication, conversations about diagnoses, cultural and religious norms and values about death and dying as well as family structures or sexual identity can impact end-of-life choice, communication and care experiences (Barrett & Wholihan, 2016; Bulut & Fikkers, 2022; Chou et al., 2015; Kwak & Haley, 2005; Napier et al., 2014; Seelman et al., 2018).

### Diversity in Dutch End-of-Life Care

In the Netherlands, palliative and end-of-life care, including end-of-life care conversations and ACP, is delivered by generalists who – if required – can be supported by healthcare professionals specialized in palliative care (Torensma et al., 2020). ACP is addressed in various toolkits, guidelines and healthcare standards (Boddaert et al., 2017; 2020; Guldemond et al., 2017; KNMG, 2019). Such aids are often the product of a collaboration between healthcare professionals and researchers, the latter providing the most recent scientific insights. In recent years, guidelines on palliative and end-of-life care have started to acknowledge the importance of adequately accounting for different aspects of diversity (e.g., ethnicity, religion, country of origin, age, socio-economic class, language, coping style, gender and sexual orientation) in palliative and end-of-life care, communication and ACP (Boddaert et al., 2020; Bulut & Fikkers, 2022).

However, the definitions of and approaches to diversity and different social identities in existing guidelines and reports vary and they do not always address how to deal with structural drivers of inequities toward the end-of-life, such as discrimination and racism. Additionally, ACP is not yet commonplace in all care environments (Van der Plas et al., 2021) and there remain differences in end-of-life care and communication preferences and outcomes between minoritized and majoritized patient groups in the Netherlands (Torensma et al., 2020).

As a concept, ‘diversity’ is often approached and defined in contradictory ways, whereby different meanings of the term are rarely made explicit which can adversely affect knowledge production in research and practice (Calasanti, 1996). To address existing disparities, more insight is needed into how diversity is currently *actually* conceptualized and engaged with in end-of-life care, communication, and research on these subjects in the Netherlands. Insight into the way in which healthcare professionals and researchers give meaning to diversity in ACP and end-of-life conversations can increase our understanding of what these definitions could mean for improving the quality of end-of-life care and communication with individual patients and communities in practice.

### Aim of the Study

The aim of this study is to explore how healthcare professionals and researchers conceptualize diversity in ACP and end-of-life conversations and how they envision diversity-responsiveness in end-of-life care and research practice in the Netherlands. We use the term diversity-responsiveness to refer to proactively enabling equal access to and quality of ACP and end-of-life conversations, interventions, and inclusive research practices for all patients. In our discussion, we reflect on how the ‘diversity discourses’ we identify could impact the provision of good and diversity-responsive end-of-life care for all patients. By reporting the results of this study, we hope to contribute to greater awareness of and reflection on how different diversity discourses may affect inclusive and equitable practices at and towards the end-of-life.

## Methods

### Study Design, Participant Selection and Sampling

We carried out an explorative, qualitative interview study among researchers and healthcare professionals in the Netherlands with the aim to investigate how diversity and diversity-responsiveness in ACP and end of-life care conversations was understood and conceptualized.

We interviewed 16 participants, 13 of which were female and three male. All were white Dutch nationals with 9–30 years of work experience. Nine participants worked as healthcare professionals (general practitioner, nurse practitioner, specialist in geriatric medicine, case manager for people with dementia, and health educator). Seven participants were experienced health and end-of-life care researchers. Participants were recruited through the authors’ professional networks by means of maximum variation purposive sampling and the snowball method (Green & Thorogood, 2014). All participants had extensive knowledge of and expertise with different aspects of end-of-life care, especially for older patients, engaging in end-of-life conversations, assessing ACP and with various aspects of diversity and inequalities in healthcare, including gender, culture, socio-economic status, religion, sexual orientation, and the intersectional overlap of these categories. All approached individuals agreed to be interviewed, however, one withdrew their consent after the interview. When someone agreed to participate, they received an information letter.

### Data Collection

MM and OÜ carried out three semi-structured face-to-face interviews and 12 phone interviews that were guided by a topic list. One face-to-face interview was conducted with two participants simultaneously. All interviews lasted between 20 and 60 minutes. Topics included questions about participants’ professional experiences with end-of-life conversations and ACP, their perspectives on whether and how diversity aspects (such as gender, ethnicity, race, religion, class, sexual orientation, and migration status) might play a role in these conversations and is currently accounted for, and which diversity competencies they believed healthcare professionals and researchers should have. While our topics focused on ACP and end-of-life conversations, many interviewees reflected on the role of diversity within the broader context of access to and delivery of end-of-life care services and research practices. In a cyclic and iterative process, topics were added to the topic list when new or unforeseen themes emerged from the information provided by the participants (Green & Thorogood, 2014). All interviews were audio recorded and transcribed verbatim.

### Data Analysis

We used thematic discourse analysis (TDA) to inductively investigate the themes and narratives underlying researchers’ and professionals’ conceptualizations of diversity and diversity-responsiveness, and to identify shared diversity discourses. Thematic discourse analysis combines thematic analysis and discourse analysis and has been used before (e.g., Eines et al., 2019; Singer & Hunter, 1999). Discourse analysis generally strives towards finding the meaning ‘beyond the sentence’ (Tannen et al., 2015) by recognizing that meaning-making is related to a wider interactional, social and cultural context (Yazdannik et al., 2017). We used TDA to explore participants’ understanding of diversity in end-of-life conversations by also considering similarities and differences between their narratives in a thematic way.

Data was analyzed in an iterative process. CK and MM read the transcripts to familiarize ourselves with the data. We then applied codes to identify the thematic and ‘interpretive systems’ (Bilmes, 1986) underlying the participants’ understanding of diversity in end-of-life conversations: we first coded the themes that emerged in each interview, and subsequently coded the argumentation structure and language use that contributed to different constructions of diversity and diversity-responsivity in ACP. The overarching codes eventually referred to the different discourses we identified. For instance, we coded participants’ discursive use of ethnicity markers such as ‘Muslim’ or ‘Turkish’, then linked this code to the argument made in the section about diversity in end-of-life conversations in which the marker occurred – e.g., ‘Muslims prefer not to talk about palliative sedation’. We then interpreted this *diversity marker-argument* dyad as a single category conceptualization of diversity (‘Muslim’ is used as a homogenous, distinct category that is relevant for diversity-responsive end-of life care). In this case we labelled the paragraph as the ‘categorical’ discourse.

We considered which discourses merged from the interviews, where they occurred, and how meaning-making regarding diversity in ACP and end-of-life conversations was constructed. Finally, we examined which sections overwhelmingly dealt with one discourse and had most argumentative depth (the ‘dominant diversity discourse’), and how the occurrence of discourses differed between participant groups. To increase reliability, CK and MM independently reread the transcripts, recoded discourses where necessary, and compared codes until intersubjective agreement was reached. All authors reflected on and provided feedback on the emerging analyses and discourses.

### Ethical Considerations

This study was conducted in accordance with quality criteria and research ethics (Green & Thorogood, 2014). To ensure confidentiality, the interview data was anonymized and coded during the handling, transport, and storing. All participants provided informed consent to participate in the study and were provided with a member check summarizing the content of their individual interview. The Medical Ethical Review Committee (METC) of the Amsterdam UMC location VUmc confirmed that the Dutch Medical Research Involving Humans Act (WMO) did not apply. Additional approval was not required.

## Results

Five discourses emerged from our data: the ‘categorical’ discourse; the ‘diversity as a determinant’ discourse; the ‘diversity in norms and values’ discourse; the ‘everyone is unique’ discourse, and the ‘anti-essentialist’ discourse. While discourses coexisted, all participants used one discourse more frequently and referred to it in more argumentative depth than others (see Table 1). Each discourse demonstrates different conceptualizations of diversity and may manifest as different approaches to conversations, care and research. The ‘categorical’ discourse refers to diversity as differences in characteristics between social groups. The ‘diversity as determinant’ discourse argues for responsiveness to outcome-related determinants, such as health literacy. The ‘diversity in norms and values discourse’ refers to the existence of social group differences in value systems that impact end-of-life conversations. The ‘everyone is unique’ discourse conceptualizes diversity as treating all patients as unique individuals. Finally, the ‘anti-essentialist discourse’ promotes awareness of intra-group differences and warns against stereotyping. We observed discursive differences between healthcare professionals and researchers. Below we will describe each discourse separately.

**Table 1.** Characteristics and dominant discourses of study participants.

	Pseudonym	Gender	Dominant discourse
Healthcare professionals			
1	Jennifer	Female	Norms and values
2	Gale	Female	Norms and values
3	Justine	Female	Norms and values
4	David	Male	Norms and values
5	Andrew	Male	Norms and values
6	Lou	Female	Everyone is unique
7	Jeanne	Female	Everyone is unique
8	Lydia^a^	Female	Norms and values
9	Alex	Male	Norms and values
Researchers			
10	Taylor	Female	Determinant
11	Sarah	Female	Determinant
12	Carmen	Female	Categorical
13	Tamsin	Female	Determinant
14	Patricia	Female	Categorical
15	Robin	Female	Categorical
16	Eleanor	Female	Categorical

a Lydia also worked as a health researcher.

### The Categorical Discourse

The categorical discourse was predominantly used by researchers in reference to evaluating end-of-life care interventions in research (i.e., equal representation in samples, constructing categories for subgroup analysis), and to describe which knowledge end-of-life healthcare professionals need about the value systems, life experiences and communication needs of social groups to provide diversity-responsive care. It was present in 14 of the 15 interviews, but most frequently appeared as dominant among researchers. Participants who used the categorical discourse discussed diversity as referring to inter-group differences, and as referring to social groups with shared characteristics that determined needs and experiences. Tamsin (researcher) explained: “I think about (…) diversity as (…) not only cultural background, but also age, sex, and possibly even sexual identity.”

Researchers used the discourse to describe the relationship between defining social categories and doing good research: producing specific knowledge about social groups was considered important to inform diversity-responsive end-of-life conversations and ACP practice. For instance, Tamsin (researcher) illustrated the importance of categorization for quantitative analysis: “Of course it is interesting for researchers to [to define diversity] because, if everyone is unique, we cannot make any predictions.” Sarah stated that while constructing social categories to enable subgroup analyses, such categories are useless when their underrepresentation in a sample results in statistical power issues.

We looked at differences concerning sex, level of education, marital status, living situation, degree of vulnerability and mental competence. And concerning the effects it might also be interesting to look at gender (…). But in our research, there is [only] a small percentage of people with non-western background, so it doesn’t really make sense to do a specific sub-group analysis. (Sarah, researcher)

Categorization was also applied to equal group representation in research samples. Lydia (healthcare professional) argued: “I think that this is a big problem that, in all of these [end-of-life] studies, women, and especially migrants. . . are underrepresented.” Similarly, Carmen (researcher) said: “People with a different [minoritized] cultural background are less represented in research studies… [on] advance care planning.” Participants argued that addressing diversity in ACP and end-of-life research means including participants of different socio-cultural-sexual characteristics to avoid biases, facilitate interpretation of variation, and predict the needs of minoritized populations.

Researchers also argued that healthcare professionals should have knowledge about potential group differences in end-of-life attitudes, wishes and experiences (e.g., concerning views on recovery, advance directives, or palliative sedation) as this allows them to adjust their communication to the needs of patients with specific group memberships Patricia (researcher) said: “What I do know is that people – Muslims, migrants – or those with other religions often do not want to know their diagnosis and prognosis, because they think that this will diminish their hope for recovery.” Eleanor (researcher) said: “I think that doctors… you can use the general term cultural competence here, and that includes that they should be able to talk about sex and gender.” Taylor (researcher) explained: “The way in which western healthcare professionals, or Dutch healthcare professionals, communicate about a poor prognosis or approaching death, many people with Turkish or Moroccan background (…) find that shocking.” Robin, using the example of lesbian and gay older people, clarified:

I also think that it is in the best interest of people in the last phase of their life that [doctors] know enough about their lifestyles so that you know which topics you should talk about (…). These are things that are really intimate but that lie outside the scope of the regular hetero-life. (Robin, researcher)

Finally, healthcare professionals used the categorical discourse only incidentally and never as the dominant discourse. Andrew, for instance, described knowledge about the value systems of particular social groups as ‘basic’ for physicians:

There are certain starting points one simply has to know, basic knowledge: about what religions say about continuing treatment, dementia, food, the gate to heaven (…). That is basic knowledge, but it is also about respect, being respectful, prevention, and [managing] expectation(s). (Andrew, healthcare professional)

### Diversity as a Determinant Discourse

Another discourse that emerged from our data is the ‘diversity as a determinant’ discourse. Participants who used this discourse argued that to achieve diversity-responsive care, individual determinants of outcomes of ACP and end-of-life conversations, such as health literacy, coping, language, communication style, cognitive capacities and illness awareness, should be considered. Paying attention to such determinants was perceived as more important than a focus on social group memberships alone, although group membership and specific determinants such as language were said to be sometimes related. This discourse appeared in 14 interviews but was only dominant among researchers (*n*=3). Tamsin (researcher) described: “I think that it [diversity] has more to do with someone’s personality-characteristics, like someone’s coping style or self-efficacy. Your ability to reflect on your disease and deal with your disease.” Participants understood determinant diversity as valuable for clinical practice to adjust ACP or end-of-life communication to patients’ language and literacy-related needs. Taylor specified what diversity-determinant responsive ACP communication looks like:

ACP has to be done differently with someone who has lower health literacy than with someone with higher health literacy. This has to do with the extent to which you have to go into detail, which jargon you use, the language, and amount of language that you use. (Taylor, researcher)

Both researchers and healthcare professionals stressed the importance of communication skills to engage in good ACP and end-of-life conversations with patients of various literacies. Arguing that healthcare professionals ought to adjust their communication style and language to meet their patients’, Jennifer (healthcare professional) stated: “‘Did you ever consider…?’, is a relatively abstract question. ‘Who shall speak for you if you cannot do so anymore?’ Is a question that also a more simple man understands.” Andrew (healthcare professional) pointed at how individual characteristics such as language and literacy could influence the end-of-life care process. He said: “We also discuss what they want with their care: how long they want to be treated, how far they want to go. And here the language barrier and health literacy is frequently a problem.”

While the diversity as determinant’ discourse was rarely used in reference to research, Sarah (researcher) described determinants of outcomes of end-of-life conversations as easy-to-use inclusion and exclusion criteria. She said: “If someone does not speak or barely speaks Dutch then they are already excluded. Because it is impossible to have a conversation about wishes and preferences (…) or to let them fill out questionnaires.”

### Diversity in Norms and Values Discourse

According to the ‘diversity in norms and values ‘discourse, which appeared in most interviews (*n*=15) but was only dominant among healthcare professionals (*n*=7), diversity entails that there can be different or even conflicting norms and values among and between patients and healthcare professionals regarding what they perceive as good end-of-life care. Participants who used this discourse problematized diversity as complicating ACP and end-of-life conversations, particularly when healthcare professionals are unaware of their own values and moral perspectives. Justine (healthcare professional) said: “We are used to our own perspectives [on what is good end-of-life care], sometimes also based on our profession. And then we don’t take into account what the other may think.” Additionally, participants stressed that being receptive to the value systems of patients is essential for diversity-responsive ACP and end-of-life conversations. This was seen as particularly important in instances when values conflict.

To ensure diversity-responsiveness of the care process, participants said that professionals must adequately deal with value-based differences between themselves and their patients. This included different moral stances toward the end-of-life, ideas about supportive care or pain relief, or willingness to talk about future care in the first place, that might come up during conversations. Lydia explained:

You can imagine that you are engaging in a conversation with someone who says: we want that my mother receives tube feeding, that you say: okay, if this is your wish, [but] that you don’t immediately confront [your patient] with your own, opposing perspective. And that [confrontation] happens a lot because many doctors in the Netherlands don’t understand or know that there are people who think about this in a fundamentally different way. (Lydia, healthcare professional)

Diversity in norms and values was seen as a source of potential moral conflict and stress, especially when the moral perspective of a patient on what constitutes good end-of-life care differed from that of the professional. Jennifer (healthcare professional) wondered: “So [it is] partially [about] my norms and values (…). How do you explain this [your different perspective]? Are you allowed to do that, is someone allowed to have their own norms [and] values?” She specified the tension she felt in such instances between her professional and her personal role: “At a deathbed (…) I feel like I am part of a very intimate family circle, where I come with my expertise and knowledge. On the other hand, also as a human being (…).” Value conflicts were perceived to elevate resistance and insecurity in discussing care preferences in the first place, out of fear of doing something wrong or being insensitive. However, participants also argued that if healthcare professionals feel that a patient’s value system is similar to theirs, mutual understanding and responsiveness could increase. Patricia (researcher) explained: “That has something to do with it being easier to understand a patient who you think is similar to you (…), for instance [a] highly educated [person] who wants to go on a sailing trip for the last time.”

Participants agreed that care professionals should specifically address and be open to norms and values that differ from their own during ACP and end-of-life conversations. Jennifer (healthcare professional) emphasized: “What is very important is (…) that you are open to what people think and that there are different ways of thinking.”

### ‘Everyone is Unique’ Discourse

The ‘everyone is unique’ discourse refers to the idea of diversity as complex and unlimited variation between people. This discourse appeared in ten interviews, was predominantly used by healthcare professionals, and dominant in three of their interviews. It was not applied to research contexts.

Participants using the ‘everyone is unique’ discourse emphasized that that diversity-responsive ACP and end-of-life communication means that all patients should be treated as unique individuals with highly personal care needs, and that patients cannot be classified according to social group membership, outcome determinants, or value systems. For instance, Jeanne (healthcare professional) argued: “Every person is different (…). You have a different person in front of you every time, and you try to attune to the person. So yes, you deal with diversity a lot because all people are different*.”* Insight into personal wishes was considered key for a good ACP process or have good end-of-life conversations. Lou (healthcare professional) said: “If the patients’ wishes are known to everyone, children professionals, then this creates the freedom to go along with the process in a natural way. Then I see less resistance.” Jennifer (healthcare professional) emphasized how such an approach required a careful choosing of questions: “There are always people for whom speaking about death is easier [than for others]. I also have to carefully explore every time what the right questions are for the individual person.” The importance of asking ‘the right questions’ to deliver diversity-responsive care appeared in many interviews. Alex (healthcare professional) for instance maintained: “By asking open and genuine questions you can uncover someone’s essence, that is diversity for me.” Jeanne explained approaching everyone as a unique person as her ‘quest’:

It is a new quest every time: who are you, who do I have in front of me, how do you interact with the next of kin (…) and how do we manage everything with each other in the time to come. This quest: it is stunning to be able to do that! (Jeanne, healthcare professional)

### The ‘Anti-Essentialist’ Discourse

The final discourse we identified is the ‘anti-essentialist’ discourse. This discourse was used by researchers and healthcare professionals to warn against stereotyping and making assumptions about sociocultural groups. The argument underlying this discourse was that for a diversity-responsive approach to ACP and end-of-life conversations in both research and clinical practice settings, it is important to be aware of intra- and inter-group differences. While this discourse was not dominant for any participant, it was present in seven of the 15 interviews and therefore included as a discourse in its own.

Both researchers and health care professionals mentioned the importance of avoiding assumptions of homogeneity, generalizations, and stereotypes. Andrew (health care professional) maintained not to translate knowledge about one group to another. In ACP practice, he said: “(…) we have to distinguish differences within migrant groups (…). Migrants are often thrown into one group, but there are different cultures.” Taylor (researcher) warned to avoid creating essentialist knowledge by attributing group characteristics to individuals. She advised: “You also have to be careful not to include anything in guidelines like ‘people with a migration background, this, people with a non-migration background, that’. You must avoid these generalizations.” Some participants, like Alex, described failure to acknowledge within-group differences as potentially problematic because it might lead to a lack of attention for individual intersections and complexity:

I think the danger lies in generalizing information. My experiences with [advance care planning] conversations (…) is that [someone] can be a Muslim who is also homosexual, just to say something, that does not say anything about Muslim culture or homosexual culture, but it says something about the individual. (Alex, healthcare professional)

Reflecting on her own experiences with research inclusion, Tamsin wondered:

I don’t know (…) if diversity is important at the group level or at the individual level. There are people who are less educated that want to participate [in research] and those that are highly educated that don’t want to participate. So, you cannot specify this for one group. (Tamsin, researcher)

Because they believed stereotypes are unavoidable, participants emphasized the importance of self-reflection to prevent biases in research and clinical practice. Jennifer talked about becoming aware of her own knowledge gaps and blind spots:

No, you really should not generalize, but everything that you do not know you often also do not see. So, the more you know, the more you see (…). So, this is about conscious competence, conscious incompetence and unconscious incompetence (…). (Jennifer, healthcare professional)

Regarding conversations with patients, Gale (healthcare professional) added: “There are many stereotypes in the things we do, and things we don’t do (…). Everybody has prejudices. You have to carefully investigate [them]*.”* Patricia (researcher) agreed: “You can’t assume that everyone [who is Muslim] thinks that speaking about death is a taboo. If you don’t regularly examine this assumption, then you continue to have this bias and to work in this way.”

## Discussion

We explored how healthcare professionals and researchers conceptualize diversity in ACP and end-of-life conversations and how they envision diversity-responsive end-of-life research and care practice. While diversity discourses often coexisted or overlapped, we distinguished five distinct discourses that we argue might have different implications for diversity-responsive end-of-life communication and care. However, despite their differences, all five discourses contain overlapping characteristics in the way in which they could either lead to the advancement of equitable and diversity-responsive end-of-life conversations and care practices, or to the further reproduction of health disparities. Below, we reflect on how conceptualizations of diversity corresponded to potential research and practice-based impacts across several themes.

### Potential Implications for Strategies Toward Equity in End-of-Life Care

First, as best illustrated by the categorical discourse, we encountered discursive patterns in which diversity was understood as similarities and differences in end-of-life perspectives and preferences based on patients’ social group membership. According to participants, such social factors should inform research designs (such as sampling procedures and choice of analytical categories) and future care conversations (such as inquiring about religious practices). This way of understanding diversity considers single categories of identity in their socio-structural context and could advance equity in end-of-life outcomes when it manifests as an effort to account for between-group variation and structural determinants. For instance, in research, accounting for gender or ethnicity enables researchers in the Netherlands to safeguard the inclusion of participants with minoritized backgrounds, employ diversity-responsive interventions and measures, and report relevant (sub)group differences. In care practice, this discourse may increase Dutch healthcare professionals’ awareness of between-group variations, and urge them to explore needs related to patients’ social identities and positions. However, if not used critically, categorical understandings of diversity could foster essentialist beliefs (McGarty et al., 1995). For instance, overemphasizing group-specific characteristics could reduce people to representatives of their group, assign group characteristics to individuals, and ignore intra-categorical complexity (McCall, 2005). Essentialism in research, care standards, and policies threatens personalized approaches and negatively impacts the physician-patient relationship; in climates hostile to notions of ‘the Other’, essentialist explanations promote harmful cultural stereotypes, and can be used as justifications for inequitable social systems (Verkuyten, 2003).

A second discursive pattern emphasized diversity as constructed group-memberships that are based on patient-centered approaches when ‘doing’ diversity and fostering diversity-responsiveness in end-of-life research and care. Examples are the ‘diversity as determinant’ and the ‘diversity in norms and values’ discourse. In the former, diversity is related to accounting for individual patient characteristics (like health literacy, language, coping, or communication style) that might influence outcomes such as uptake of palliative care services and engagement in ACP (Volandes et al., 2008; De Vries et al., 2019). In the latter, diversity is conceptualized in the context of healthcare professionals’ attentiveness to the variation in norms and values (i.e., moral diversity or pluralism) that exist between either themselves and their patients, or within their patient population. Both discourses concern characteristics of individual patients (i.e., determinants like health literacy or specific values or value systems) and assign group memberships on the basis of these characteristics. A potential benefit of framing diversity as differences in health determinants might be that Dutch healthcare professionals can improve outcomes by adjusting their care to the specific educational, socioeconomic, and cultural context of patients and their families. Further, specifically regarding the ‘diversity in norms and values’ discourse, healthcare professionals’ awareness of moral pluralism and its role in patient-care provider communication might advance equitable care at the individual level. It has, for instance, been argued that physicians should be respectful of their patient’s wishes and avoid imposing their own values (Fulmer et al., 2018), and that addressing conflicting values between dominant and minoritized world views is an important first step towards cultural inclusivity in ACP and end-of-life decision-making (Johnstone & Kanitsaki, 2009). The latter can include structured reflection on norms and values through dialogical practices like moral case deliberation, which has been argued to foster knowledge, skills and mutual moral learning between and among healthcare professionals and patients (Stolper et al., 2016).

However, a downside of understanding diversity in this way might be that systemic factors, such as poverty or institutional racism, are left unaccounted for in ACP and end-of-life conversations. In research, individual characteristics are easily used as exclusion criteria in research because low (health) literacy and language barriers complicate recruitment, implementation, and evaluation of interventions, effectively keeping perspectives of already marginalized individuals out of the research cycle. The perpetuation of this process can reinforce epistemic injustice, the situation in which the voices of those that are difficult to measure are not understood or disregarded (Fricker, 2007). Furthermore, it is unclear how to operationalize diversity on the basis of moral variation in the research process, for instance in the recruitment of participants or analytical techniques. In practice, demanding absolute responsiveness to the norms and values of others may cause moral challenges or distress for Dutch healthcare professionals, if this forces them to act in opposition with their own moral perspective on good care. Furthermore, in their encounters with patients, healthcare professionals might find that sole focus on personal moral differences perpetuates rather than resolve value conflicts.

Third, as illustrated in the ‘everybody is unique discourse’, another conceptualization of diversity refers to the notion that to achieve diversity-responsiveness, all patients should be approached as individuals with unique wishes, needs and contexts. Focusing on the individuality of patients might increase equity in daily practice, by allowing professionals to communicate about and provide care that is specifically catered to a specific person. Further, the relationship between viewing patients as unique and quality of care has been stressed in literature on personalized care planning (e.g., Edwards et al., 2017). However, both in research and practice, an uncritical understanding and conceptualization of diversity as uniqueness can limit awareness of the role of structural inequalities in access to good end-of-life conversations and prevent the integration of a health justice stance in guidelines and policies. Overlooking group-based disparities in ACP and end-of-life care may perpetuate the underservicing and exclusion of already minoritized social groups in end-of-life care practice and research (e.g., Cloyes et al., 2018; Evans & Ume, 2011; Wicks et al., 2018). This concern builds on the challenges voiced in response to the ‘diversity as determinant’ or ‘diversity in norms and values’ discourses.

A final discursive pattern and conceptualization of diversity is immanent in the ‘anti-essentialist’ discourse, which specifically acknowledges the importance of reflecting on biases and privileges. As argued by participants who used this discourse, harmful overgeneralizations cause biases, and hinder personalized approaches ACP and end-of-life care conversations. Therefore, diversity-responsiveness means avoiding stereotyping, recognizing intra-group differences, and reflexivity. These perceptions are in line with anti-essentialism as “(…) an emancipatory discourse in the challenge of hegemonic representations, the fixity of identities and oppressive relations” (Verkuyten, 2003, 373). In addition, they align with the tenets of intersectionality theory: the idea that individuals have multiple intersecting social identities associated with either societal privilege or disadvantage (e.g., experiences or racism, sexism, homophobia, etc.) (Kassam et al., 2020; McCall, 2005). Intersectional approaches could play an important role in addressing disparities in end-of-life care, because they aid in recognizing inequalities in, for instance, care access and quality of care, that are experienced by people from multiple historically oppressed groups (Corpora, 2021). Applying an intersectional lens to end-of-life conversations in the Netherlands might direct how access is widened, which questions are asked, and how a patient’s sociocultural context is considered in the end-of-life care process. Therefore, intersectional knowledge and skills (e.g., awareness of essentialism, the ability to recognize how social identities interact to shape needs, or critical self-reflexivity) are essential additions to practices associated with any of the other diversity discourses. However, it remains unclear how to practically align anti-essentialist understandings of diversity with current evidence-based research designs and methodologies (e.g., Randomized Controlled Trials) that construct medical knowledge based on population averages, and with diagnostic methods that depend on such knowledge to classify and predict individual outcomes.

### Fostering Awareness

Based on the above reflections, we argue that there is no single way of conceptualizing diversity that is universally beneficial and always leads to equitable approaches to end-of-life conversations and care (see also Sue & Dhindsa, 2006). Instead, in theory, any of the diversity discourses might encourage both inclusive or inequitable practices. Therefore, our study highlights the importance of being aware of one’s own ‘diversity discourse’, and the potential consequences of its application in research and care. Healthcare professionals and researchers in the Netherlands ought to reflect on their own normative diversity perspectives, and how those shape their ACP and end-of-life conversations, care practices and research. In addition, at the institutional level, awareness of the real-life impact of a dominant discourse might encourage administrations and care organizations to continuously monitor whether diversity policies indeed lead to more equitable health care. This also includes reflecting on how diversity is conceptualized in guidelines on end-of-life care and ACP. For instance, whether such guidelines acknowledge if and how group memberships are assigned, the importance of critical consciousness, or whether they offer concrete insight into how to address multiple, intersecting identities**.** Critically engaging with diversity and reflecting on social impact is particularly important as ‘diversity’ and ‘inclusion’ can sometimes be treated as important, yet abstract policy buzzwords. For instance, while many organizations formulate diversity policies, the performativity of such documents increases the risk that they become mere paper trails with little actual effect or accountability (Ahmed, 2007; Jansen et al., 2021).

### Study Limitations and Future Research

This study has several limitations. First, our participant sample was homogenous in terms of ethnic background, nationality and educational level. The overrepresentation of healthcare professionals and researchers from the dominant culture asks that our findings are considered in relation to the social positions of our participants (white Dutch, highly educated), which limits their theoretical transferability. Additionally, this study was exploratory in nature and we included both healthcare professionals and researchers in our sample. Future research should include a larger sample size with a broader representation of social identities, or focus on both professions individually, in order to critically compare similarities and differences between these groups in a meaningful way. Furthermore, additional data is needed to gain a deeper understanding of the actual relationship between discourse, practices and outcomes, e.g., through observational studies or mixed methods approaches, or through the inclusion of patient perspectives. Examining the experiences of patients and their next of kin with healthcare professionals’ diversity approaches may generate a better understanding of the entanglement of professionals’ discursive practices and quality of end-of-life conversations. Lastly, various definitions of end-of-life care (Gysels et al., 2013) and interventions and approaches to ACP and end-of-life conversations (Goswami, 2021) exist. This plurality is also evident in our participant’s discourses. Future research should explore manifestations of healthcare professionals’ diversity discourses across and within various contexts in which different definitions and approaches to ACP and end-of-life conversations exist.

## Conclusion

This article provides insight into the discourses healthcare professionals and researchers use to conceptualize diversity, and into how their conceptualization of diversity may inform their understanding of diversity-responsive approaches to ACP and end-of-life conversations in research and care practice. A thematic discourse analysis yielded five diversity discourses. We argue that each discourse has the potential to advance equity and social justice through facilitating diversity-responsive approaches in end-of-life research and care. However, uncritical use of these discourses might also reproduce existing disparities. We argue that awareness and reflection on personal diversity discourses and their potential impact is an ethical imperative for researchers and professionals concerned with ACP and end-of-life conversations, and for all whose understanding of diversity, individually and collectively, has the power to counter health inequities and promote good end-of-life care in policy and practice.
